# Editorial Notices

**Published:** 1874-05

**Authors:** 


### EDITORIAL NOTICES.

### CRUSHED WHITE WHEAT.

No more pernicious custom exists than that of sacrificing the most nutritious portion of the wheat in the process of converting it into flour. The grain of wheat, chemically, is a miniature man. Before using it for food we bolt out the brains, bones and nerves—and if the missing elements were not supplied from other articles of diet, the most deplorable consequences would ensue. When those king structures, the bones, brains and nerves are poorly nourished, other organs, notably the lungs, show the general blight first; and it is a significant fact that consumption is always preceded and accompanied by a species of amenia, which the rejected portion of wheat, could it be properly digested and assimilated, is precisely adapted to cure. If bread made, as Nature intended it should be, from all the constituents of wheat, was as universally used as that from its white emasculated residue, called "superfine flour," a few generations would see consumption and scrofula, with their legion of kindred evils, less prevalent. In looking for a reliable article of food, containing all the nutritious elements of the best wheat, we have been much pleased with that manufactured by F. E. Smith & Co., Atlantic Flour Mills, Nos. 18, 20 and 22 Hamilton avenue, Brooklyn, N. Y.

### CAUSES OF CONSUMPTION.

Whatever tends to produce fatigue or exhaustion, whether from mental or physical labor, decreases the quantity of carbonic acid gas exhaled from the lungs. Excessive fatigue, as well as want of exercise—apparently opposite causes—are equally predisposing tendencies to consumption; the former, by exhaustion; the latter, by stagnation of the functional and muscular action.

Every kind of activity—intellectual, passional, locomotive or generative; all causes of depression; such as grief, overwork, excesses, fretting, insufficient food, rapid growth, pregnancy, nursing, long illness, wasting from fevers, protracted convalescence, etc.—is followed by an undue waste of the phosphorus of the system, as is proved by an increase in the excretions of the "phosphates." If this waste is not arrested by rest, nutrition, and a resupply of the phosphoric element, nervous debility and impoverishment of the blood are inevitable consequences—both of which are marked characteristics of pulmonary disease.

To supply this waste the administration of the hypophosphates has come into great favor among educated physicians. It is a matter of prime importance that the remedy should be properly prepared and chemically pure. That prepared and sold by J. Winchester & Co., 36 John street, New York, is the best known, and believed to be the most reliable.

### FUMIGATING AND DISINFECTING.

While all agree upon the necessity of a free use of disinfectants under certain circumstances, very little regard has heretofore been had to economy in their use. Carbolic acid, which is the most important, is also the most expensive of disinfectants. If, by a simple and cheap mechanical contrivance, as much foul air can be disinfected with one ounce of material as with one pound, used in the ordinary way, a very important result is attained. We have been experimenting with "Outwater's Eureka Vaporizer" with the most satisfactory results. We can vouch for the advantages claimed for this instrument. For particulars see advertisement of E. H. Gouge, in this Journal.

